# Sucessos e Desafios no Enfrentamento das Doenças Cardiovasculares no Brasil: Viver Mais e Melhor

**DOI:** 10.36660/abc.20210589

**Published:** 2021-08-09

**Affiliations:** 1 Universidade Federal de Minas Gerais Faculdade de Medicina Departamento de Clínica Médica Belo HorizonteMG Brasil Departamento de Clínica Médica, Faculdade de Medicina, Universidade Federal de Minas Gerais, Belo Horizonte, MG - Brasil.; 2 Faculdade de Saúde e Ecologia Humana Faculdade de Medicina Belo HorizonteMG Brasil Faculdade de Medicina, Faculdade de Saúde e Ecologia Humana, Belo Horizonte, MG - Brasil.; 3 Universidade Federal de Minas Gerais Hospital das Clínicas Centro de Telessaúde Belo HorizonteMG Brasil Serviço de Cardiologia e Cirurgia Cardiovascular e Centro de Telessaúde, Hospital das Clínicas, Universidade Federal de Minas Gerais, Belo Horizonte, MG – Brasil.

**Keywords:** Sucessos, Desafios, Epidemiologia, Doenças Cardiovasculares, Câncer

As doenças cardiovasculares (DCV) e o câncer representam as maiores causas de morte no Brasil e no mundo. Tendo em vista tamanha relevância epidemiológica, o artigo “*Taxas de Mortalidade por Doenças Cardiovasculares e Câncer na População Brasileira com Idade entre 35 e 74 Anos, 1996-2017*”,^1^ por meio da análise dos dados de mortalidade das Estatísticas Vitais do DATASUS (Sistema de Informação de Mortalidade – SIM), apresenta o perfil de mortalidade por esses grupos de doenças, discute sua evolução entre 1996 e 2017, e estima a contribuição futura dessas causas de morte, caso as tendências sejam mantidas. Os principais achados incluem a maior contribuição atual das DCV para a mortalidade no Brasil, porém com redução gradual de suas taxas padronizadas por idade. Esta tendência não ocorre para as taxas de mortalidade por câncer – que permanecem estáveis – de forma que em poucos anos o câncer se tornará a principal causa de morte no país.

As DCV e o câncer, apesar de possuírem diferentes etiopatogenias, compartilham fatores de risco (FR), como tabagismo, obesidade, diabetes, consumo excessivo de álcool e baixo nível socioeconômico. Dessa forma, a manutenção da saúde cardiovascular ideal é inversamente proporcional à incidência de câncer.^2^ Assim, torna-se importante entender as tendências de exposição da população a esses FRs comuns ao longo das últimas décadas, quando as mudanças no estilo de vida derivadas da urbanização e o envelhecimento populacional contribuíram para a incidência e mortalidade elevadas de ambas as doenças.^3^ Com essas informações em mente, Mansur e Favarato analisaram os dados de mortalidade de homens e mulheres por todas as causas, por DCV, doenças isquêmicas do coração (DIC), doenças cerebrovasculares (DCbV) e câncer neste período de 21 anos.

As taxas de mortalidade proporcionais por DCV (30%) e câncer (20%) foram responsáveis por metade das mortes entre 1996 e 2017. Nesse período, a taxa de mortalidade padronizada para idade por DCV teve redução de 38%, dado que está em consonância com as estimativas do estudo *Global Burden of Disease (GBD) 2017* publicados por Malta et al.,^4^ que demonstrou uma diminuição de 34,8% de 2000 a 2017. O estudo GBD tenta, ao processar os dados primários de mortalidade do país através de modelos que incluem correções para subnotificações e redistribuição de códigos *garbage*, minimizar as limitações do SIM - como as disparidades na cobertura e na proporção de causas mal definidas de óbito, historicamente maior nos estados menos desenvolvidos.^4^

Os autores também observaram que as taxas de mortalidade por DCV padronizada por idade são menores entre as mulheres e que a diminuição da mortalidade foi mais expressiva nesse grupo. Martins et al.,^5^ frente a achados semelhantes, atribuiu ambas as tendências à maior adesão das mulheres ao rastreamento e prevenção dessas doenças na atenção primária à saúde (APS), além da proteção hormonal que sabidamente retarda a mortalidade por DCV.^5^ DIC e DCbV foram responsáveis por 57% das mortes por DCV, com as taxas de mortalidade por DCbV mostrando redução mais acentuada, possivelmente devido ao melhor reconhecimento, tratamento e controle da hipertensão arterial sistêmica (HAS) nesse período,^6^ fator de risco mais fortemente relacionado à DCbV do que a DIC – esta mais associada a fatores metabólicos do que a DCbV, os quais tiveram tendências desfavoráveis no período.^7^^,^^8^

O padrão descendente da taxa de mortalidade por DCV tanto em homens quanto em mulheres no Brasil está intrinsecamente relacionado à implantação de políticas públicas para controle dos FR – como aquelas voltadas para o controle do tabagismo ou que permitiram acesso ao tratamento da HAS – da implementação do sistema de atendimento de urgências e emergências em 2003, além de melhorias e expansão da rede de Atenção Básica no país.^6^ Tais medidas promovem hábitos saudáveis, permitem o diagnóstico e tratamento precoce das DCV agudas e crônicas, além do controle de seus determinantes – pilares do enfrentamento da DCV. Apesar desse relativo sucesso, é importante salientar que as taxas de mortalidade por DCV ainda são altas, como citam Mansur e Favarato,^1^ e o país ainda tem grandes desafios: a redução desigual das taxas de mortalidade, menor nos estados brasileiros menos desenvolvidos e entre os homens, o crescente número de mortes pelo crescimento e envelhecimento populacional, além do aumento da prevalência de obesidade e suas consequências metabólicas adversas.^6^^,^^8^

Em relação ao câncer, o estudo observou que não ocorreram variações significativas na taxa de mortalidade padronizada para idade na população geral entre 1996 e 2017. Isso aconteceu devido ao aumento de 5,8% da mortalidade em mulheres (Variação percentual anual média [VPAM]=0,3%, p=0,2), apesar da redução significativa de 3,7% entre homens (VPAM=-0,1%, p<0,001). É importante notar que, entre as regiões do país, há grandes diferenças nos padrões de mortalidade. Nas regiões Norte e Nordeste, por exemplo, são mais comuns neoplasias relacionadas a infecções – característica comum em países de baixa e média renda – enquanto que as outras regiões do Brasil apresentam padrão semelhante ao de países de alta renda, com cânceres associados ao envelhecimento e a condições crônicas.^9^^,^^10^ Além disso, as regiões Norte e Nordeste apresentam tendências de aumento na mortalidade por câncer até 2030, enquanto que nas outras regiões, as tendências são estáveis ou de diminuição,^11^ revelando como a maior mortalidade, independente da causa, está intimamente relacionada à pobreza, que atua desfavoravelmente em várias frentes: educacionais, nutricionais, e de acesso a diagnóstico e tratamento.^10^

Diante do exposto, o aumento proporcional da mortalidade por câncer é esperado na medida em que a mortalidade por DCV reduz, já que as causas de morte são competitivas. Portanto, sendo a morte inexorável, vale destacar outro dado revelado por Mansur e Favarato: a redução mais expressiva da mortalidade por todas as causas e, principalmente por DCV, nos grupos etários mais jovens, revelando uma diminuição da mortalidade prematura no Brasil nas últimas décadas,^11^ um dos Objetivos de Desenvolvimento Sustentável propostos pela Organização Mundial de Saúde para 2030.^12^ Em suma, os dados do presente estudo mostram avanços, mas reforçam que desafios perenes e novos requerem a implementação e renovação de políticas públicas que promovam o enfrentamento das DCV e do câncer como prioridades no cenário da saúde no país, para que os brasileiros possam viver mais e melhor.
