# Supplementary material for: Sucessos e Desafios no Enfrentamento das Doenças Cardiovasculares no Brasil: Viver Mais e Melhor
Source: Arq Bras Cardiol. 2021 Aug 9;117(2):341–2. [Article in Portuguese] doi: 10.36660/abc.20210589 (PMC8395809; doi:10.36660/abc.20210589)
Supplement: Translation

# Translation: Successes and Challenges in the Management of Cardiovascular Disease in Brazil: Living Longer and Better

**Affiliations:** 1 Belo HorizonteMG Brazil Departamento de Clínica Médica, Faculdade de Medicina, Universidade Federal de Minas Gerais, Belo Horizonte, MG - Brazil.; 2 Belo HorizonteMG Brazil Faculdade de Medicina, Faculdade de Saúde e Ecologia Humana, Belo Horizonte, MG - Brazil.; 3 Belo HorizonteMG Brazil Serviço de Cardiologia e Cirurgia Cardiovascular e Centro de Telessaúde, Hospital das Clínicas, Universidade Federal de Minas Gerais, Belo Horizonte, MG – Brazil.

**Keywords:** Successes, Challenges, Epidemiology, Cardiovascular diseases, Cancer

Cardiovascular diseases (CVDs) and cancer are the main causes of death in Brazil and worldwide. Considering this epidemiological relevance, the article “Mortality Rates from Cardiovascular Diseases and Cancer in the Brazilian Population aged 35 to 74 Years, 1996-2017”,^1^ through the analysis of mortality data from the DATASUS Vital Statistics (Mortality Information System – SIM), depicts the mortality profile of these groups of diseases, discusses their evolution between 1996 and 2017, and estimates the future contribution of these causes of death, if the trends are maintained. The main findings include the greater current contribution of CVDs to mortality in Brazil, but with a gradual reduction of their age-standardized rates. This trend did not occur for cancer mortality rates – which remain stable – so that, in a few years, cancer will become the main cause of death in the country.

CVDs and cancer, despite having different etiopathogeneses, share risk factors (RF), such as smoking, obesity, diabetes, excessive alcohol consumption and low socioeconomic status. Therefore, maintaining an optimal cardiovascular health is inversely proportional to the incidence of cancer.^2^ Thus, it is important to understand the population exposure trends to these common RFs over the last decades, when lifestyle changes resulting from urbanization and population aging contributed to the high incidence and mortality of both diseases.^3^ With this information in mind, Mansur and Favarato analyzed mortality data from all causes for men and women, CVD, ischemic heart disease (IHD), cerebrovascular disease (CbVD) and cancer over this 21-year period.

Proportional mortality rates from CVD (30%) and cancer (20%) accounted for half of the deaths between 1996 and 2017. During this period, the age-standardized mortality rate from CVD decreased by 38%, which is in line with the estimates of the Global Burden of Disease 2017 study published by Malta et al.,^4^ which showed a decrease of 34.8% from 2000 to 2017. The GBD study tries, when processing the country's primary mortality data through models that include corrections for underreporting and redistribution of garbage codes, to minimize the limitations of SIM – such as disparities in coverage and in the proportion of ill-defined causes of death, historically higher in less developed states.^4^

The authors also observed that age-standardized CVD mortality rates are lower among women and that the decrease in mortality was more significant in this group. Martins et al.^5^ considering similar findings, attributed both trends to women's greater adherence to screening and prevention of these diseases in primary health care (PHC), in addition to the hormonal protection that is known to delay mortality from CVD among women.^5^

IHD and CbVD were responsible for 57% of CVD deaths, with CbVD mortality rates showing a more pronounced reduction, possibly due to better identification, management and control of systemic arterial hypertension (SAH) in this period,^6^ a risk factor more strongly related to CbVD than IHD – that is more associated with metabolic factors than CbVD, which showed unfavorable trends in the period.^7^^,^^8^

The downward pattern of CVD mortality rate in both men and women in Brazil is intrinsically related to the implementation of public policies for the control of RFs – such as those aimed at smoking control or those allowing access to SAH treatment – the implementation of urgent and emergency care system in 2003, as well as improvements and expansion of the Primary Care network in the country.^6^ These measures promote healthy habits, allow for the early diagnosis and treatment of acute and chronic CVDs, in addition to the control of its determinants – the pillars of CVD treatment. Despite this relative success, it is important to note that CVD mortality rates are still high, as mentioned by Mansur and Favarato,^1^ and the country still faces major challenges: the uneven reduction in mortality rates, which is lower in less developed Brazilian states and among men, the growing number of deaths due to population growth and aging, in addition to the increased prevalence of obesity and its adverse metabolic consequences.^6^^,^^8^

Regarding cancer, the study observed that there were no significant variations in the age-standardized mortality rate in the general population between 1996 and 2017. This occurred due to the 5.8% increase in mortality among women (Mean Annual Percentage Change [MAPC] =0.3%, p=0.2), despite the significant reduction of 3.7% among men (MAPC=-0.1%, p<0.001). It is important to note that, across regions of the country, large differences in mortality patterns are observed. In the North and Northeast regions, for instance, infection-related neoplasms are more usual – a common characteristic in low- and middle-income countries – while other regions of Brazil show a pattern similar to that of high-income countries, with cancer associated with aging and to chronic conditions.^9^^,^^10^ Additionally, the North and Northeast regions show increasing trends in cancer mortality until 2030, while the trends are stable or decreasing in the other regions,^11^ showing how higher mortality, regardless of the cause, is closely related to poverty, which acts unfavorably on several fronts: educational, nutritional, and regarding access to diagnosis and treatment.^10^

Given the abovementioned facts, a proportional increase in mortality from cancer is expected as the mortality from CVD decreases, since the causes of death are competitive. Therefore, since death is unavoidable, it is worth highlighting other information disclosed by Mansur and Favarato: the more significant reduction in mortality from all causes and, especially, from CVDs, in the younger age groups, disclosing a decrease in premature mortality in Brazil in recent decades,^11^ one of the Sustainable Development Goals proposed by the World Health Organization for 2030.^12^

In short, the data from the present study show advances, but reinforce that perennial and new challenges require the implementation and renewal of public policies that promote the fight against CVDs and cancer as priorities in the health scenario in the country, so that Brazilians can live longer and better.
